# The Maimonides Heritage: Discovery and Propagation of Medical Knowledge

**DOI:** 10.5041/RMMJ.10340

**Published:** 2018-07-30

**Authors:** Rafael Beyar, Karl Skorecki, Shraga Blazer

**Affiliations:** 1 Director, Rambam Health Care Campus, Haifa, Israel; 2 Director, Medical & Research Development, Rambam Health Care Campus, Haifa, Israel; 3 Editor-in-Chief, Rambam Maimonides Medical Journal, Haifa, Israel

Dear Friends and Colleagues,

Since its launch in 2010, *Rambam Maimonides Medical Journal* (*RMMJ*) has focused on its mission of expanding the knowledge base of medicine, science, humanity, and ethics throughout the world, flavored by the salt of the 850-year-old philosophy of Rabbi Moshe Ben Maimon—known by the Hebrew acronym “RAMBAM” or, more commonly, as Maimonides.

Maimonides’ monumental works on Jewish law, ethics, and medical practice remain classics. He was a famous doctor in strong demand, and his writings testify to how he cared for patients—from morning to night. Despite his full workload studying the Torah and practicing medicine, he understood that great knowledge was accompanied by great responsibility—the vast medical knowledge he had acquired could not be kept to himself nor his immediate colleagues. Hence, he was one of the few Jewish physicians of his time to write medical works for the benefit of future medical practitioners and patients worldwide. In all, he wrote 12 medical volumes, including *Treatise on Poisons and Their Antidotes*, *Treatise on Asthma*, *Treatise on Hemorrhoids*, *Glossary of Drug Names*, and *Treatise on Cohabitation*. His medical books became a cornerstone for medical studies in Asia, Africa, and Europe in later generations, and they remain remarkably relevant today.

The July issue of *RMMJ* marks our eighth year of publication, concomitant with the 80th anniversary of Rambam Health Care Campus, which also bears the Hebrew name of Maimonides and is proud to sponsor the journal. If the flavor of Maimonides permeates both the hospital and this publication, then the number eight is the spice of this particular issue. The number eight, according to Kabbalah, “is symbolic of an entity that is one step above the natural order, higher than nature and its limitations”^1^—put simply, this number points to that which seems to be impossible.

Today, medicine, science, and research are breaking barriers, and achieving depths of understanding and discovery that were once deemed beyond comprehension. New treatments and therapies are being discovered in an exponential manner; we are looking deeply into the genome and displaying the inner workings of our body with advanced imaging modalities, leveraging the laws of physics to the benefit of human health, with the trajectory of lifespans now extending to more than the 100 years. Quantities of medical data and knowledge are outgrowing our mental analytical capacity and thus necessitating new classes of big data analytic tools. Rambam Health Care Campus is world renowned for many of its accomplishments along these lines.

Likewise, this humble publication, *RMMJ*, has been indexed in PubMed Central after barely three years and is listed in the Thomson Reuters Emerging Sources Citation Index. To the best of our knowledge, *RMMJ* is the only such international peer-reviewed open-access journal that is sponsored by a hospital and supported solely by donations.

With Maimonides as the inspiration, this should not be surprising. As a Rabbi, philosopher, and physician, Maimonides also strived to achieve the seemingly impossible. He experienced persecution for his ethnicity and faith and became an exile amongst a people that were ambivalent, to say the least, regarding all he represented. Highly respected in his own community, Maimonides did not believe that helping his “own” was enough. His works and life influenced prominent Arab and Muslim philosophers, scientists, and physicians. His works were translated into a number of languages, and he has had a lasting influence on Judaism as we know it today. His perspective was simple—knowledge is meant to be shared with all who may benefit from it.

Established in 1938 as the Governmental Hospital in Haifa under the British Mandate, the medical center was transferred to the local and then governmental authorities upon the establishment of the State of Israel in 1948. Inspired by the teachings and relevance of Maimonides, it was renamed “Rambam” in 1952. Yet, the impossible was already happening within the hospital’s wards and laboratories. Long before the State’s establishment, the hospital had become accustomed to caring for both Jewish and Arab victims of trauma—casualties of war and terror. Eventually, wartime experience would make Rambam Health Care Campus the most experienced hospital in Israel in treating trauma patients—with the highest percentage of trauma survivors. Such experience was costly, paid for by the blood of patients and the post-traumatic stress disorder experienced by casualties and staff.

The spirit of Maimonides prevailed, and that cost was transformed from a liability into a benefit that, today, extends far beyond this small nation’s borders. In 1999 Rambam’s Teaching Center for Trauma, Emergency, and Mass Casualty Situations (MCS) was established with the express goal of disseminating hard-gained experience to all who need it. Since its establishment more than 3,000 health-care professionals and administrators from more than 80 nations have received training from Rambam’s Teaching Center for Trauma to enable them to establish trauma and MCS systems in their own institutes and countries. Medical teams have been dispatched to every corner of the world as part of rescue and training teams for a variety of natural and manmade disasters. Research into post-traumatic stress disorder among hospital staff has gained high visibility worldwide.

Today, Rambam Health Care Campus is the tertiary health-care provider for all of Northern Israel, serving a population of a 2.5 million people, and is the only Level 1 trauma care provider in the region. The medical center has spearheaded innovative medical procedures and discovery, developed hospital-based information technology systems, and partnered with major medical device companies, helping to develop medical technologies that have become transformative worldwide. Naming just a few examples, the CARTO 3D Anatomical Mapping System (Biosense Webster, Ltd., a Johnson & Johnson Company, Haifa, Israel), the PillCam (Given Imaging, Yokneam, Israel), and GE Global Healthcare’s cardiac SPECT/CT system were all developed or first investigated for clinical use in Haifa, at Rambam Health Care Campus.

The pervasive spirit of Maimonides in turn has become the driving force behind *RMMJ*. Established in 2010, the vision of the editorial board was to create a platform that would play a major role in the dissemination of up-to-date medical and scientific data worldwide.

The result has been a well-received journal with an international distribution, publishing peer-reviewed papers from around the world. *Rambam Maimonides Medical Journal* is a general medical journal, featuring a wide variety of content—over the years we have published review articles written in India,^2^ original research by established Canadian teams,^3^ and papers looking at the impact of Jewish values and their importance to medical professionalism. Reviews, original research, perspective pieces, unique overviews on Jewish contributions to medicine, the presentations of Nobel Laureates, and many others constitute the more than 290 papers published to date in *RMMJ*.

*Rambam Maimonides Medical Journal* represents a humble effort to contribute also to the propagation of medical humanities and bioethics in the wider world community. Medical and scientific knowledge must no longer be reserved for the upper echelons of academia or heavily funded publications. We aim to achieve the “metaphysical eight”—the impossible—by establishing a new paradigm for scholarly publications, and we believe we are succeeding. As of this issue, we have more than 17,000 subscribers, and 65,224 readers from 177 countries and territories; 23.8% of our readers are from Israel—the rest (76.2%) represent the global interest and need for what *RMMJ* so uniquely offers. There have been more than 331,500 unique downloads of our content.

We would like to close with this short true story about the impact of our *RMMJ* on a doctor traveling in South America. He was traveling in a very-high-altitude area and was not feeling well. He realized his symptoms were those of acute mountain sickness; however, he could not recall what treatment was necessary. Far from medical care or a consultant, but near a computer, he asked his wife to purchase a good article on this condition. She did an internet search and was surprised to find that a leading article in the search results was available for free from an Open Access journal—*Rambam Maimonides Medical Journal*! They did not have to pay, and she read the article to him at an altitude exceeding 3,000 meters. In it he found exactly what he needed to do to make the rest of his trip comfortable and healthy. On his return he wrote to us, sharing how thankful he was that we had made this publication freely available to all who needed it. This is but one small example of how knowledge can save lives.

Life-changing impact is what every medical professional wants to achieve. This issue of *RMMJ* therefore celebrates the 80th anniversary of Rambam Health Care Campus and the eighth anniversary of our publication by presenting you, our readership, with some outstanding papers from Rambam physicians—past and present. Each paper underwent an originality check, standard peer review, the arduous process of revision, and final copy editing.

This issue is a remarkable tribute to our beginnings, our hope for the future, and the spirit of Maimonides, which we hope will continue to inspire us to achieve the impossible.

## Abbreviations

RMMJ: Rambam Maimonides Medical Journal.
